# The limits to growth – energetic burden of the endogenous antibiotic tropodithietic acid in *Phaeobacter inhibens* DSM 17395

**DOI:** 10.1371/journal.pone.0177295

**Published:** 2017-05-08

**Authors:** Sabine Eva Will, Meina Neumann-Schaal, Raymond Leopold Heydorn, Pascal Bartling, Jörn Petersen, Dietmar Schomburg

**Affiliations:** 1 Technische Universität Braunschweig, Department of Bioinformatics and Biochemistry and Braunschweig Integrated Center of Systems Biology (BRICS), Braunschweig, Germany; 2 Leibniz Institute DSMZ-German Collection of Microorganisms and Cell Cultures, Braunschweig, Germany; Universite Paris-Sud, FRANCE

## Abstract

*Phaeobacter inhibens* DSM 17395, a model organism for marine *Roseobacter* group, was studied for its response to its own antimicrobial compound tropodithietic acid (TDA). TDA biosynthesis is encoded on the largest extrachromosomal element of *P*. *inhibens*, the 262 kb plasmid, whose curation leads to an increased growth and biomass yield. In this study, the plasmid-cured strain was compared to the wild-type strain and to transposon mutants lacking single genes of the TDA biosynthesis. The data show that the growth inhibition of the wild-type strain can be mainly attributed to the TDA produced by *P*. *inhibens* itself. Oxygen uptake rates remained constant in all strains but the growth rate dropped in the wild-type which supports the recently proposed mode of TDA action. Metabolome analysis showed no metabolic alterations that could be attributed directly to TDA. Taken together, the growth of *P*. *inhibens* is limited by its own antibacterial compound due to a partial destruction of the proton gradient which leads to a higher energetic demand. The universal presence of TDA biosynthesis in genome-sequenced isolates of the genus *Phaeobacter* shows that there must be a high benefit of TDA for *P*. *inhibens* in its ecological niche despite the drawback on its metabolism.

## Introduction

Due to its nutritional versatility *Phaeobacter inhibens* DSM 17395, originally isolated from seawater at the Spanish Atlantic coast in Galicia [1, 2] is commonly used as a model system to investigate the physiology and metabolic capabilities of marine bacteria living in nutrient-rich environments [3–5]. In addition to its 3.2 Mb chromosome, *P*. *inhibens* DSM 17395 carries three extrachromosomal elements of 65, 78 and 262 kb size, which were classified as chromids based on a relatively synonymous codon usage [6, 7] but will be further on designated as “plasmids” for reasons of simplicity. The largest plasmid encodes, in addition to proteins involved in metabolic pathways and exopolysaccharide formation, proteins involved in the formation of the antibacterial compound tropodithietic acid (TDA) [8–10]. In a recent study by Zhao et al. (2016) [11], the probiotic effect of *P*. *inhibens* in aquacultures is attributed to TDA in killing unwanted marine pathogens [12–14].

TDA is supposed to act by disturbing the proton gradient due to an exchange of extracellular protons for cytoplasmic cations [15]. Furthermore, Wilson et al. (2016) [15] proposed a counteracting resistance mechanism consisting of an active proton export in *P*. *inhibens* via the γ-glutamyl cycle. They claimed that three genes of *P*. *inhibens* DSM 17395 (*tdaR1* to *tdaR3*) are necessary for conferring an increased TDA tolerance (S2 File). However, experiments to select TDA-resistant or -tolerant strains of *Escherichia coli*, *Pseudomonas aeruginosa* or *Staphylococcus aureus* were not successful and furthermore, *Salmonella typhimurium* mutants with nonfunctional efflux pump and porin genes showed the same TDA susceptibility as wild-type strains [16]. Porsby et al. (2011) [16] explained an observed inhibition of the TDA-producing *Phaeobacter* strain 24–7 by a low TDA concentration with the prediction that TDA-producing strains express their resistance mechanism only during active TDA-production, which was not active in their experimental setup. In process-controlled bioreactors, *P*. *inhibens* DSM 17395 showed a faster growth (3.2-fold higher growth rate), a ~40% higher growth yield and a significant higher carbon usage efficiency when the 262 kb plasmid encoding the TDA biosynthesis genes was missing [8]. The TDA biosynthetic gene cluster (S2 File) consists of 6 genes coding for proteins involved in the biosynthesis, 1 transcriptional regulator and 3 genes which were described as resistance genes by Wilson et al. (2016) [15]. The biosynthesis of TDA and catalyzed steps are proposed by Brock et al. (2014) [10]. They proposed the biosynthesis to start from phenyl-acetate. The TDA production is induced by N-3-hydroxydecanoylhomoserine lactone (R-3OHC_10_-HSL) and also by TDA itself [9].

In this study, we cultivated different transposon mutants of genes that are essential for the TDA biosynthesis and compared them to the 262 kb plasmid-cured mutant and the wild-type of *P*. *inhibens* DSM 17395 to analyze the impact of endogenously produced TDA on *P*. *inhibens* itself. The data clearly show that the absence of TDA is the major reason for the higher biomass yield of mutant strains lacking a functional TDA biosynthesis. Furthermore, our analysis documents that with increasing TDA concentration the wild-type strain uses a higher share of oxygen to keep up its proton gradient. Whereas the mutants deplete the whole of the available sources the wild-type decreases its growth rate to 35% compared to ½ OD_max_ despite the fact that substrate is still available. We could not detect any increase of respiratory activity as a defensive counteraction against TDA on metabolic level.

## Materials and methods

### Strains and growth conditions

Strains used in this work are listed in Table 1. Transposon mutagenesis was performed and mutants were checked for homogeneity as previously described [17] (S1 File).

**Table 1:** Strains used in this work.

Strain	genetic modification	Position of the transposon	Reference
DSM 17395	wild-type strain	–	DSMZ^1^
Δ262-kb	curation of the 262 kb plasmid in DSM 17395	–	[8]
*tdaA*	*tdaA* transposon mutant of DSM 17395	107502+	this work
*tdaB*	*tdaB* transposon mutant of DSM 17395	106916-	this work
*tdaC*	*tdaC* transposon mutant of DSM 17395	105936+	this work
*tdaE*	*tdaE* transposon mutant of DSM 17395	104157-	this work

^1^ DSMZ = German Collection of Microorganisms and Cell Cultures, Braunschweig, Germany

All cultivations were performed in a defined salt water medium [18] with 1% of casamino acids as carbon source. Casamino acids were obtained from Merck (Darmstadt, Germany), prepared in a 5x stock solution and adjusted to pH 6.5 prior to sterile filtration. All cultivations were conducted at 150 rpm and 28°C in an orbital incubator (Certomat BS-1, orbit 50 mm; Sartorius, Göttingen, Germany). Growth was followed spectrophotometrically (OD_600nm_) and gravimetrically. TDA formation was followed optically by the brownish complex in the medium [19]. The absorption of the TDA-iron complex was measured at 398 nm [9]. Additionally, we determined absorption at 302 nm which is the main absorption maximum for TDA (S3 File, S3 Fig).

For growth curves and metabolome analysis, 500 mL Erlenmeyer flasks with 3 baffles were used.

For determination of the oxygen uptake rate, the cultures were also grown in 500 mL baffled Erlenmeyer flasks with 100 mL medium. On the bottom inside each flask was an optical oxygen sensor spot (SP-PSt3-YAU-D7-YOP; PreSens, Regensburg, Germany) attached. The incubator was equipped with an online oxygen monitoring device (Shake Flask Reader; PreSens, Regensburg, Germany). The measurement of the dissolved oxygen (DO) in the culture medium is based on fluorescence quenching [20] and the amount of fluorescence signal decrease is correlated to the oxygen concentration in the solution. Data acquisition and analysis was carried out with Shake Flask Reader Software v2.0.0 (PreSens, Regensburg, Germany) and the oxygen uptake rate (OUR) was automatically calculated. The specific OUR was calculated with the growth rate during the linear growth phase.

### Medium exchange experiment

Medium exchange experiments were performed in 250 mL Erlenmeyer flasks with 3 baffles. Cells were grown to ½ OD_max_ and harvested by centrifugation. The culture supernatants were sterile filtered and the cells were resuspended in either their own or the culture supernatant of another strain before the resulting growth rate was determined. For a detailed workflow description of the medium exchange experiment see results and discussion.

### Amino acid quantification and metabolome analysis

Amino acids were quantified in samples taken at half-maximal and maximal growth of 4 biological cultivations. Samples were prepared and analyzed by a 1260 Infinity HPLC system equipped with a fluorescence detector (Agilent Technologies, Waldbronn, Germany) and a Poroshell HPH-C18 separation column (4.6 x 100 mm, particle size 2.7 mm; Agilent Technologies) as described previously [21]. Samples for metabolome analysis were taken of 4 biological cultivations at half-maximal growth and each cultivation was analyzed in triplicates. Cell lysis, preparation and metabolome analysis was performed as described previously [18, 22] with some minor modifications: ethanol was replaced by methanol and 500 μL of the polar phase were dried prior to derivatization.

## Results and discussion

### Growth behavior of mutants with a missing or incomplete TDA biosynthesis

**Fig 1:**
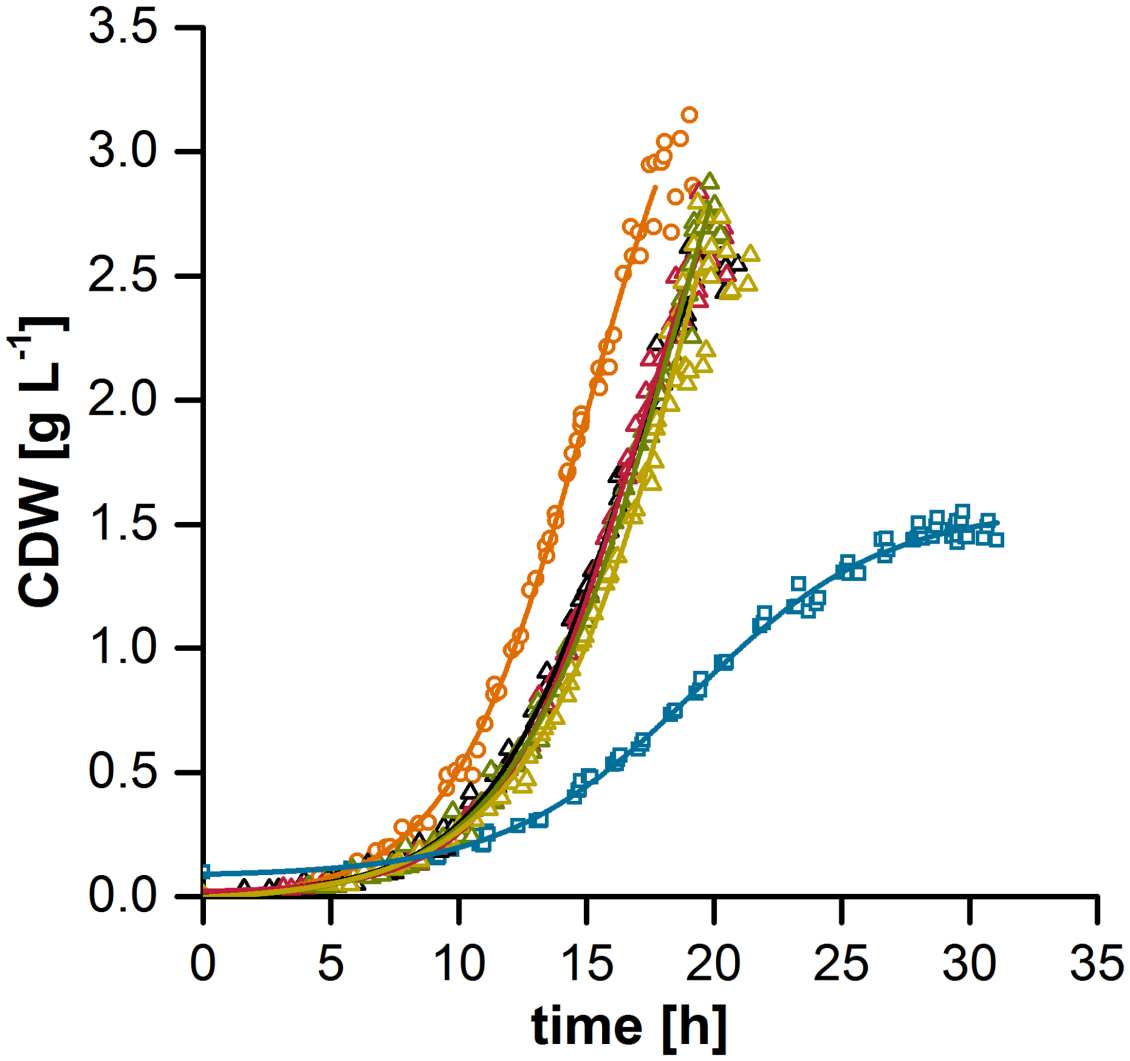
Growth curves of *P*. *inhibens* DSM 17395 wild-type and tropodithietic acid (TDA)-free mutants on casamino acids. Blue squares/line: wild-type, orange circles/line: plasmid-cured mutant Δ262-kb, black triangles/line: *tdaA* transposon mutant, red triangles/line: *tdaB* transposon mutant, green triangles/line: *tdaC* transposon mutant, ocher triangles/line: *tdaE* transposon mutant. Cell dry weight (CDW) was determined of overall 7 biological replicates from 2 independent cultivations. Time points of biological replicates were corrected for the lag phase to the replicate with the shortest lag phase. Due to the specific growth behavior, growth curves were only fitted until CDW_max_ according to the Boltzmann model using OriginPro2015 software, further time points were measured.

Four transposon mutants (*tdaA*, *tdaB*, *tdaC*, *tdaE*), the plasmid-cured mutant (Δ262-kb) and the corresponding wild-type *P*. *inhibens* DSM 17395 were cultivated with casamino acids as the carbon source. In absence of a functional TDA biosynthesis, the maximal biomass concentration was reached after approximately 20 h of growth due to the depletion of any available carbon source while the wild-type showed a drastically reduced growth rate, lower final biomass yield and entered the stationary growth phase after 30 h despite a remaining high concentration of amino acids (total amount 6.04 ± 1.44 mM, corresponding to 16% of the initial concentration) in the medium (Fig 1, S1 Table). Overall the different transposon mutant strains showed an approximately 11% lower biomass yield than the Δ262-kb mutant strain while the growth rates during the linear growth phase remained similar. In contrast the biomass yield of the wild-type was reduced by approximately 35% compared to the transposon mutants and by approximately 41% compared to the Δ262-kb mutant strain. The difference in amino acid depletion for plasmid-cured mutant and wild-type was already observed by Trautwein et al. (2016) [8]. The lower growth yield for the wild-type was explained by its slow replication rate or the metabolic burden for plasmid carriage and production of exopolysaccharides. However, our growth experiment showed that the growth inhibition of the wild-type is directly connected to the TDA biosynthesis as its biosynthesis is encoded by the 262 kb plasmid. The data suggest that only a minor effect can be attributed to other metabolic pathways encoded by the 262 kb plasmid or the metabolic costs of plasmid carriage.

### Medium exchange between wild-type and TDA-free mutants

**Fig 2:**
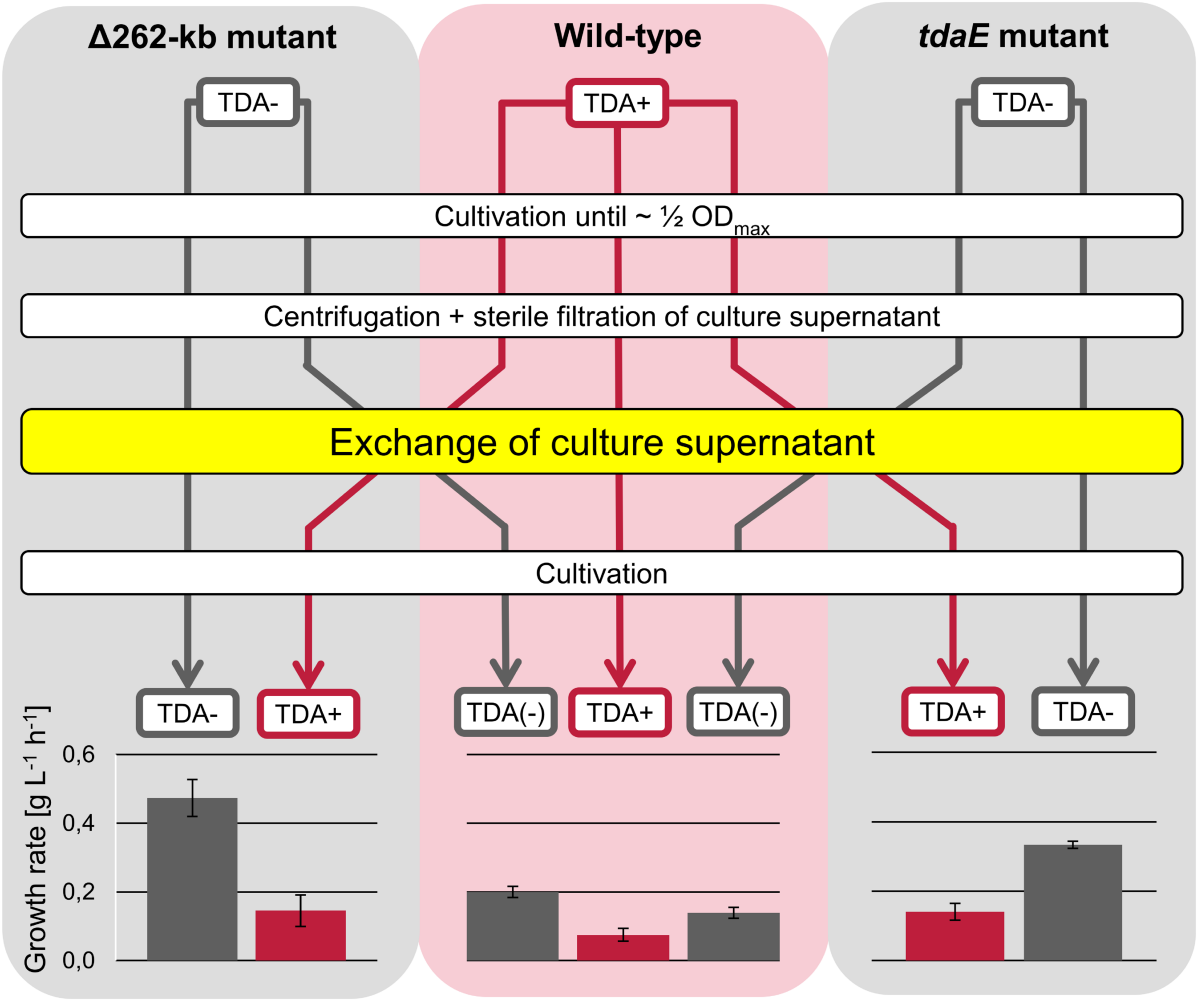
Medium exchange experiment between the wild-type and tropodithietic acid (TDA)-free mutants. TDA-free mutants and the wild-type were simultaneously grown until about ½ OD_max_. The cells of TDA-free mutants and wild-type were separated from their medium and resuspended in the supernatant of the wild-type and TDA-free mutant, respectively. The reference cultures were resuspended in its own supernatant. Subsequently, the cultivation was continued. The linear growth rates (g L^-1^ h^-1^) were determined by linear fit of the cell dry weight (CDW) values in the first hours after the exchange procedure. Arrows indicate the medium (red: with TDA, grey: without TDA). The background shows the strain (red: TDA-producing strain, grey: strain with missing or incomplete TDA biosynthesis).

To exclude the possible costs of TDA biosynthesis itself as the source for the decrease of growth yield, a selected transposon mutant (*tdaE*) and the plasmid-cured mutant (Δ262-kb) were applied in medium exchange experiments with the wild-type (Fig 2). We selected the transposon mutant of the *tdaE* gene encoding an acyl-CoA dehydrogenase which catalyzes the first step of the *tda* operon in the TDA biosynthesis [10] to avoid side effects of precursors on the metabolism. The presence of TDA in the supernatant of the wild-type under the given growth conditions was verified by measurement of absorption at 302 and 398 nm (S3 File and S3 Fig). A growth inhibition was observed for both mutants after transferring the cells into the culture supernatant of the wild-type. Furthermore, the wild-type showed an increased growth rate when transferred to the TDA-free supernatant of the mutants. As a control experiment, we performed an exchange of the culture supernatant between the two TDA-free mutants to check whether the process of changing the medium or any difference in the amino acid concentration has an influence on the growth rate. Growth rates after this medium exchange were similar to the growth rates before the change (S1 Fig).

The growth inhibition caused by the culture supernatant of the wild-type showed that a produced compound of the wild type is the cause for the inhibition effect. The control experiment with an exchange between transposon and plasmid-cured mutant approved that the growth inhibition is directly connected to TDA.

### Constant oxygen uptake rates allow only lower growth rates in presence of TDA

The resistance mechanism proposed by Wilson et al. (2016) [15] assumes that glutamate is available in the medium and γ-aminobutanoic acid (GABA) would be excreted. However, glutamate is one of the preferred amino acids and is rapidly consumed by *P*. *inhibens* [5]. In contrast to the proposed resistance mechanism of Wilson et al. (2016) [15], we could not detect any accumulation of γ-aminobutanoic acid in the culture supernatant. *P*. *inhibens* shows only a decreased growth rate pointing to a high energy demand. A partial destruction of the proton gradient means that more respiratory activity is required to obtain the necessary ATP regenerated by ATP hydrolase. In previous studies, an almost 2-fold higher oxygen consumption per cellular dry weight was observed for the wild-type compared to the plasmid-cured mutant in processed-controlled bioreactors [8]. Thus, we measured the oxygen saturation in Erlenmeyer flasks and determined the oxygen uptake rate (OUR) in relation to the biomass at different time points of growth (Table 2, S2 Table, S2 Fig). All mutants showed a constant growth rate until CDW_max_ while the wild-type strain showed a decreasing growth rate with proposed accumulation of TDA in the culture supernatant, correlating with the brown color. In contrast, the OUR remained comparable at approximately 1000 L h^-1^ g^-1^ for the wild-type and the mutants until the transition phase. In the context of the mode of action of TDA [15], this indicates that at constant oxygen uptake rates per cell dry weight, the presence of TDA leads to a slower growth rate due to lower ATP yield without any decrease of respiratory activity.

**Table 2:** Oxygen uptake rates and growth rates.

strain	Time point	Relative growth rate [%]	OUR/CDW [L g^-1^ h^-1^]
DSM 17395	½ OD_max_	100 ± 6	1091 ± 61
	¾ OD_max_	82 ± 12	1033 ± 63
	~ OD_max_	35 ± 17	893 ± 125
Δ262-kb	½ OD_max_	100 ± 1	999 ± 49
	¾ OD_max_	114 ± 4	1030 ±13
	~ OD_max_	110 ± 8	859 ± 138
*tdaE*	½ OD_max_	100 ± 8	1075 ± 147
	¾ OD_max_	115 ± 14	1072 ± 144
	~ OD_max_	111 ± 15	1038 ± 112

Shown are relative growth rates to the growth rate at ½ OD_max_ and oxygen uptake rate (OUR) based on the cell dry weight (CDW) for the wild-type strain DSM 17395 and the mutant strains Δ262-kb and *tdaE* determined for 3 biological replicates each. 3 time points were chosen for the comparison (½ OD_max_, ¾ OD_max_ and shortly before reaching OD_max_). For detailed information see experimental procedures, S2 Table and S2 Fig.

### Metabolome analysis of wild-type and TDA-free mutant strains

**Fig 3:**
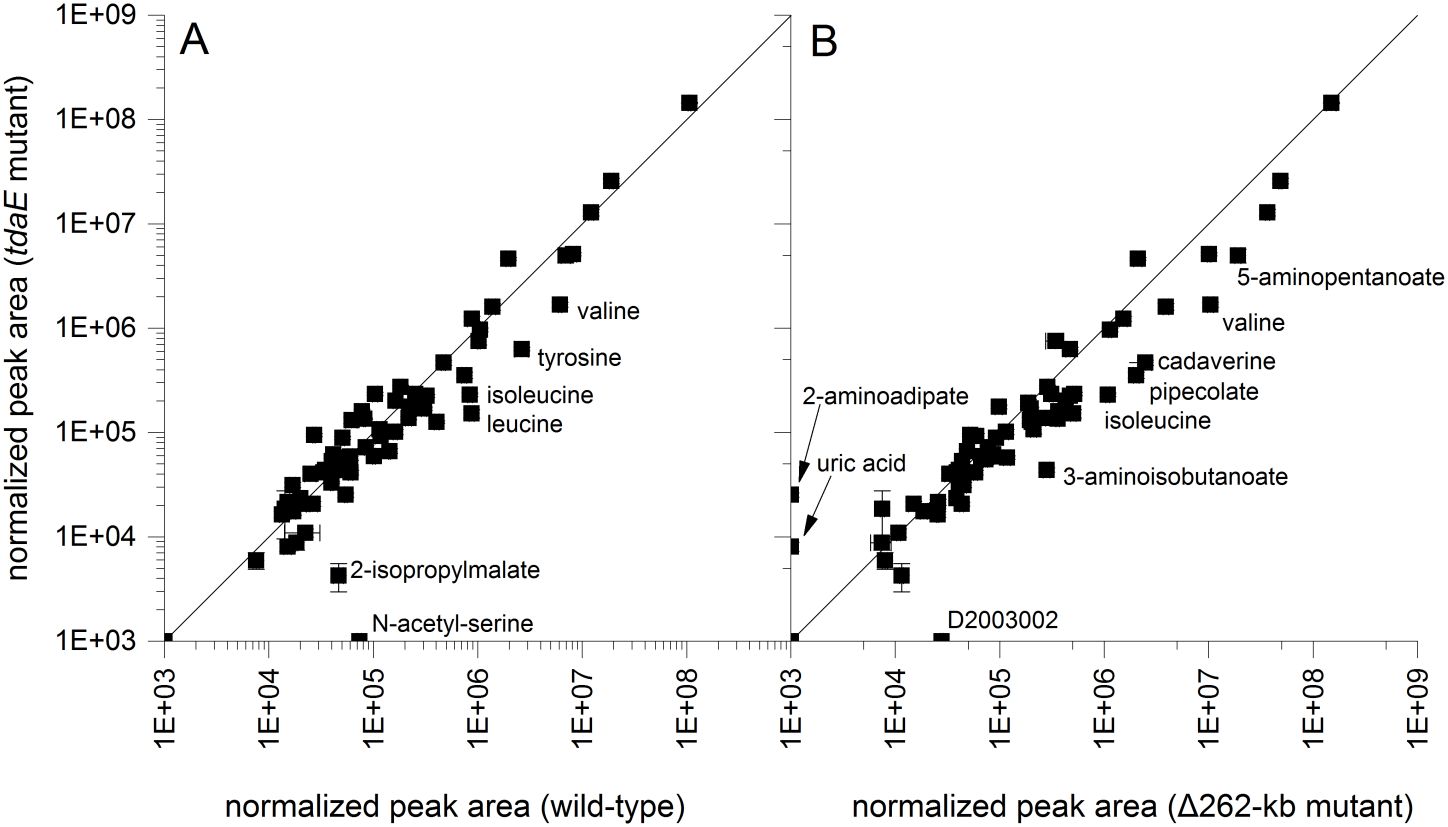
Comparison of logarithmized normalized peak areas of tropodithietic acid (TDA)-free mutants. TDA-free mutants and the wild-type were simultaneously grown until about ½ OD_max_. Shown are metabolites found in cell extracts after cultivation on casamino acids in comparison of the *tdaE* mutant to the wild-type (A) and the Δ262-kb mutant (B). Values represent the average of four independent experiments. Error bars represent the standard error between the four experiments.

As we could not detect a higher respiratory activity, we performed a metabolome analysis of the wild-type and the two mutant strains (*tdaE* and Δ262-kb) to analyze possible differences in their metabolism (Fig 3, S3 and S4 Tables). Only few changes in metabolites could be observed for the *tdaE* mutant compared to the wild-type strain (Fig 3A) in cells at half-maximal biomass yield. N-acetyl-serine was only detectable in the wild-type strain and may be involved in regulatory functions. Increased levels of valine and tyrosine in the wild-type in comparison to the *tdaE* mutant may be attributed to the different amino acid utilization at half-maximal growth as both amino acids were less or not used by the wild-type strain at this stage of growth (S5 Table). From half-maximal growth on, isoleucine and leucine are less consumed by the wild-type which may explain the observed increased intracellular levels at half-maximal growth (S1 Table). 2-Isopropylmalate is directly linked to increased levels of valine. These experiments show that the inhibition by TDA has only minor direct effects on metabolic processes except slower biosynthesis rates. Furthermore, we compared the metabolome of the *tdaE* transposon mutant with the plasmid-cured strain (Fig 3B). Again, we detected differences in isoleucine, valine and its degradation product 3-aminoisobutanoate. Other metabolites which are altered between the strains can be attributed to genes encoded by the 262 kb plasmid involved into the proposed lysine degradation pathway via pipecolate in *P*. *inhibens* [23]. One gene (locus tag: PGA1_262p02210) shows sequence similarity to the pipecolate oxidase in *Pseudomonas putida* KT 2440 (40% identity, E-value 4E-97) [24]. Thus, concentrations of several metabolites of the different lysine degradation pathways are altered: cadaverine, pipecolate, 5-aminopentanoate and 2-aminoadipate. The unidentified compound D200302 shows the typical mass spectrum and retention index of a modified sugar and can be attributed to alterations due to the absence of the exopolysaccharide biosynthesis proteins encoded by a gene cluster on the 262 kb plasmid (locus tags: PGA1_262p00050 to PGA1_262p00130) with sequence similarities to the succinoglycan biosynthesis of *Rhizobium meliloti* 1021 [25] (S6 Table). Overall, none of the changes in the metabolome can be directly attributed to the influence of TDA.

## Conclusion

In this work we demonstrate that the presence of the antibacterial compound TDA in *P*. *inhibens* DSM 17395 is associated with a dramatic impact on the growth of the bacterium. The TDA-producing wild-type strain shows a reduced growth rate and—more importantly—a significantly lower biomass yield at the same oxygen uptake rate as the mutants, we could not observe any counteraction neither on metabolic level nor on the respiratory activity. Accordingly, our data support the effect of TDA on the proton motive force proposed by Wilson et al. (2016) [15]. Moreover, we show that TDA is the major reason for the observed growth restriction by the 262 kb plasmid previously reported by Trautwein et al. (2016) [8]. Only a minor part can be attributed to other genes encoded by the plasmid. As TDA biosynthesis genes encoding plasmids can be found in all *Phaeobacter* species [6, 26, 27], TDA must provide a significant advantage to *P*. *inhibens* in a competition for nutrients which compensates the high energetic costs.

## Supporting information

S1 Fig Medium exchange experiment between the mutant strain Δ262-kb and *tdaE* (control experiment). Experiment was performed analog to the main experiment (Fig 2). Supernatant of the plasmid-cured mutant was changed with the supernatant of the transposon mutant. The growth rate was determined directly after the exchange procedure. For detailed workflow see Fig 2 and experimental procedure. Grey bars: reference, white bars: medium exchange.
(PDF)

S2 Fig Measurement of oxygen uptake in erlenmeyer flasks with the shake flask reader. Shown are the growth curves with corresponding dissolved oxygen saturation (DO) and oxygen uptake rate (OUR) for the wild-type strain DSM 17395 and the mutant strains Δ262-kb and *tdaE*. The growth curves were fitted until CDW_max_ according to the Boltzmann model using OriginPro2015 software. For determination of growth rates at the chosen time points, the fit was differentiated to get the slope at this point. For detailed information on the oxygen uptake measurement see experimental procedures.
(PDF)

S3 Fig Verification of TDA production. Shown are growth curves with corresponding absorption at 302 nm (TDA) and 398 nm (iron-complexed TDA) for the wild-type strain DSM 17395 and as controls for the mutant strains Δ262-kb and *tdaE*.
(PDF)

S1 File Authenticity of transposon mutants. (PDF)

S2 File TDA biosynthetic gene cluster and proposed biosynthesis pathway. (PDF)

S3 File Absorption spectrum of TDA, culture supernatants of the wild-type strain DSM 17395 and the mutant strains Δ262-kb and *tdaE* and of the casamino acids medium. (PDF)

S1 Table Amino acid consumption upon entering the stationary phase in the wild-type strain DSM 17395 and the mutant strains Δ262-kb, *tdaA*, *tdaB*, *tdaC* and *tdaE*. Analyzed were the concentrations of all amino acids present in casein hydrolysate (casamino acids) in the stationary growth phase via HPLC-FLD (see Experimental Procedures). Shown are the amino acids concentrations (mmol L^-1^) of each replicate and the calculated mean values with corresponding deviation.
(XLSX)

S2 Table Measurement of oxygen uptake in erlenmeyer flasks with the shake flask reader. The table contains all chosen OD values with corresponding cell dry weight (CDW) values for the wild-type strain DSM 17395 and the mutant strains Δ262-kb and *tdaE*. Furthermore, it contains the corresponding dissolved oxygen saturation (DO) and oygen uptake rate (OUR) data as well as the OUR based on CDW. For detailed information on the oxygen uptake measurement see experimental procedures.
(XLSX)

S3 Table Metabolome analysis of the wild-type strain DSM 17395 and mutant strains *tdaE* and Δ262. Shown are the normalized peak areas of each replicate and the calculated mean values with corresponding standard error of all identified metabolites detected at least in one of the strains. nd: not detectable.
(XLSX)

S4 Table Metabolome analysis of the mutant strains *tdaE* and Δ262 compared to the wild-type strain DSM 17395. Shown are all identified metabolites detected at least in one of the strains. Fold changes are calculated between the indicated strains (strain A/strain B in the column heading) including corresponding standard error. nd: metabolite not detectable in strain A; +: metabolite detectable in strain A but not in strain B; -: metabolite neither detectable in strain A nor in strain B.
(XLSX)

S5 Table Amino acid consumption at ½ OD_max_ in the wild-type strain DSM 17395 and the mutant strains Δ262-kb and *tdaE*. Analyzed were the concentrations of all amino acids present in casein hydrolysate (casamino acids) at ½ OD_max_ via HPLC-FLD (see Experimental Procedures). Shown are the amino acids concentrations (mmol L^-1^) of each replicate and the calculated mean values with corresponding deviation.
(XLSX)

S6 Table Putative exopolysaccharide biosynthesis gene cluster. Shown are blastp hits against the genome of *Rhizobium meliloti* 1021 with corresponding E-values and identities. Furthermore, the sequences were analyzed with InterPro to predict domains.
(XLSX)
