# Supplementary figures and images for: The limits to growth – energetic burden of the endogenous antibiotic tropodithietic acid in Phaeobacter inhibens DSM 17395
Source: PLoS One. 2017 May 8;12(5):e0177295. doi: 10.1371/journal.pone.0177295 (PMC5421792; doi:10.1371/journal.pone.0177295)

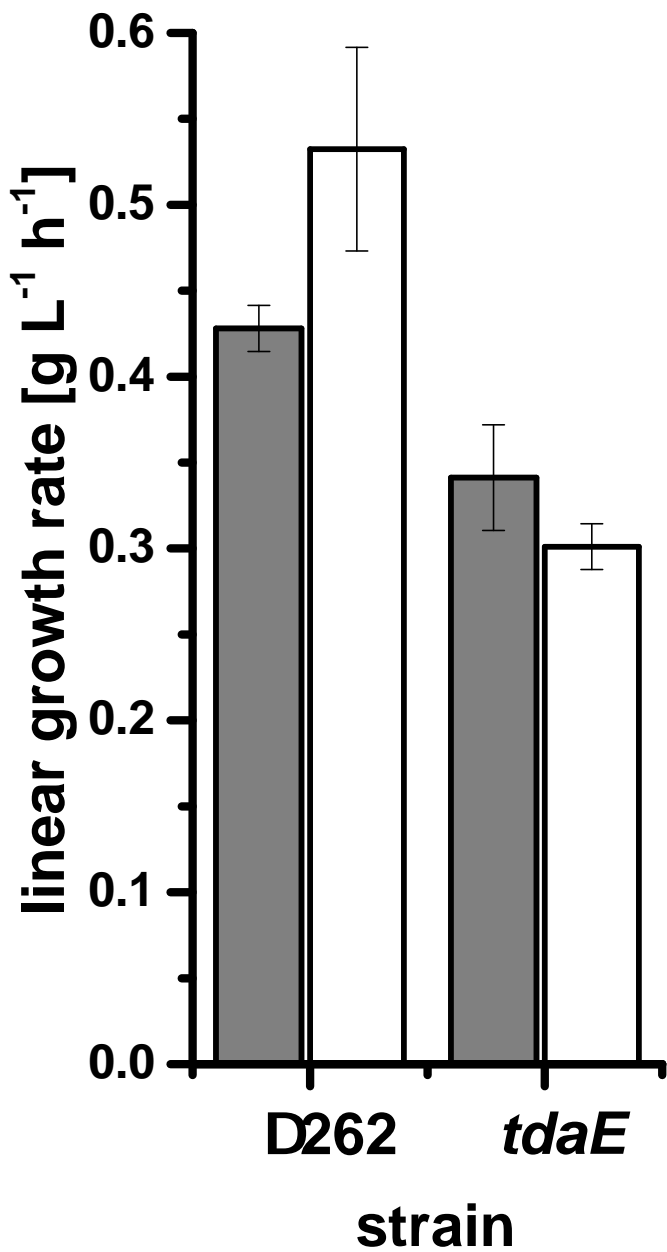

Supplement: S1 Fig — Experiment was performed analog to the main experiment (Fig 2). Supernatant of the plasmid-cured mutant was changed with the supernatant of the transposon mutant. The growth rate was determined directly after the exchange procedure. For detailed workflow see Fig 2 and experimental procedure. Grey bars: reference, white bars: medium exchange. (PDF) [file pone.0177295.s001.pdf]

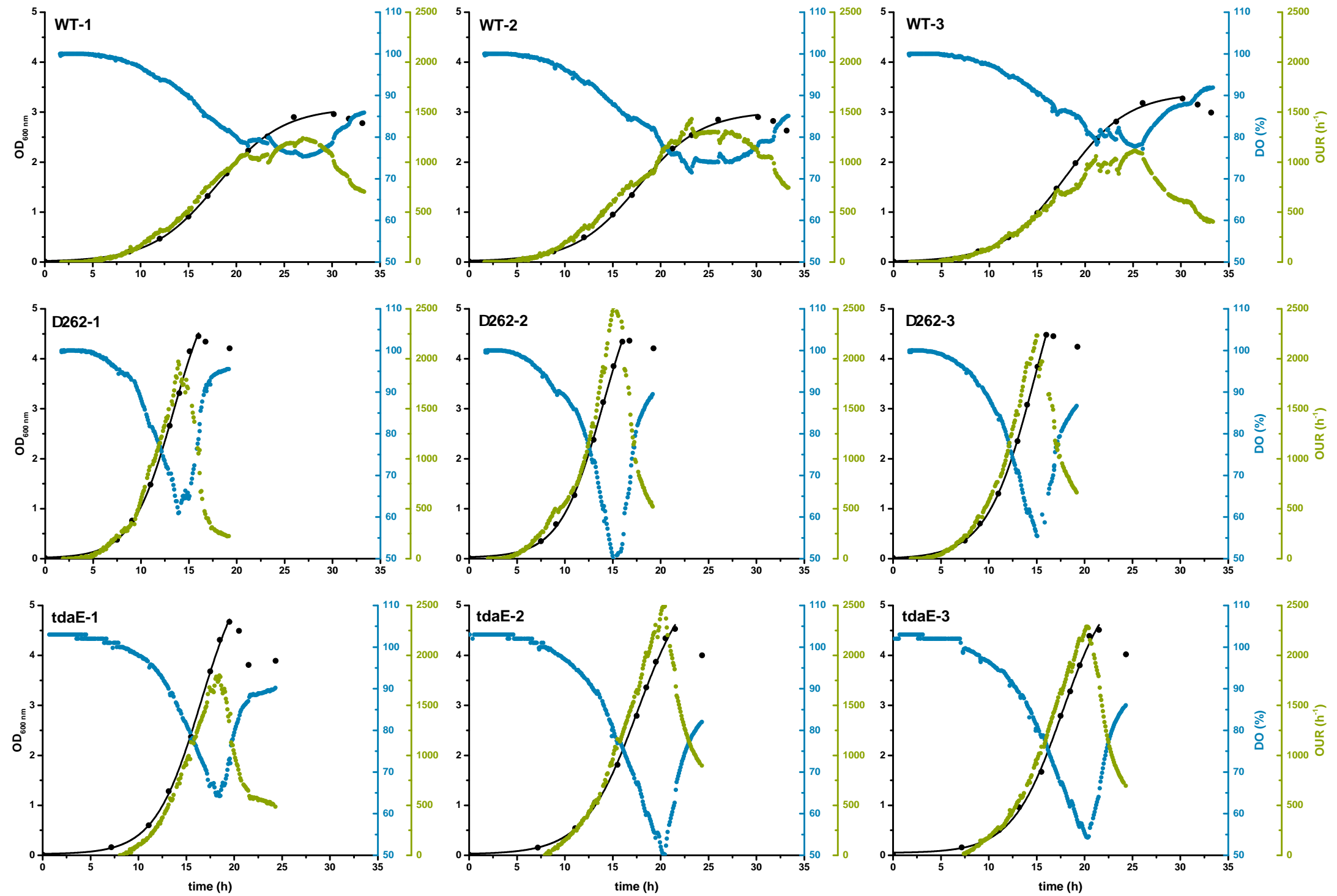

Supplement: S2 Fig — Shown are the growth curves with corresponding dissolved oxygen saturation (DO) and oxygen uptake rate (OUR) for the wild-type strain DSM 17395 and the mutant strains Δ262-kb and tdaE. The growth curves were fitted until CDWmax according to the Boltzmann model using OriginPro2015 software. For determination of growth rates at the chosen time points, the fit was differentiated to get the slope at this point. For detailed information on the oxygen uptake measurement see experimental procedures. (PDF) [file pone.0177295.s002.pdf]

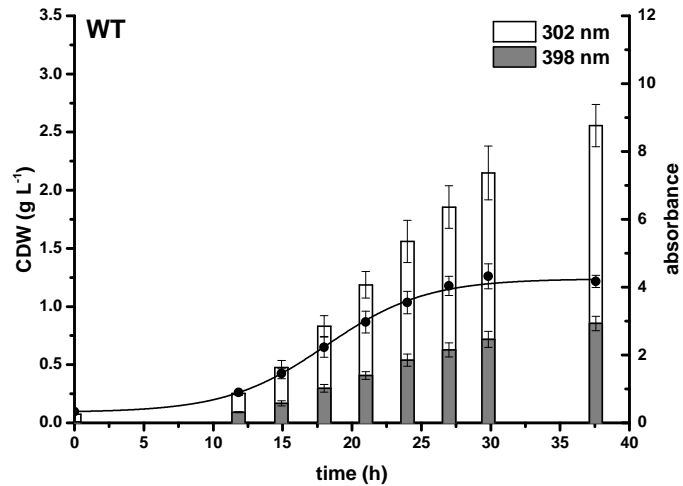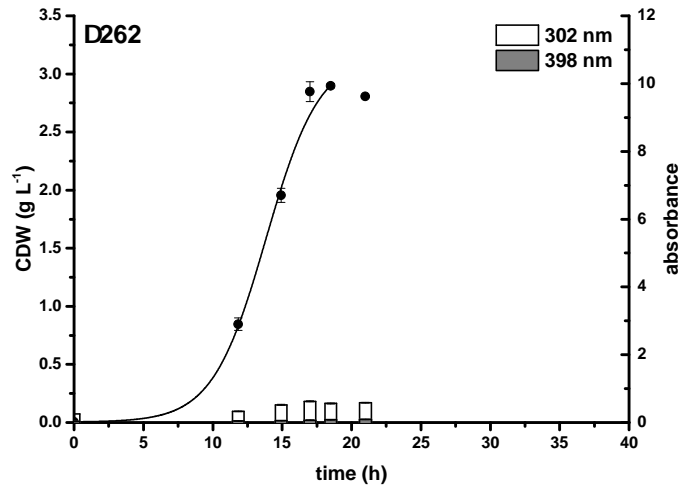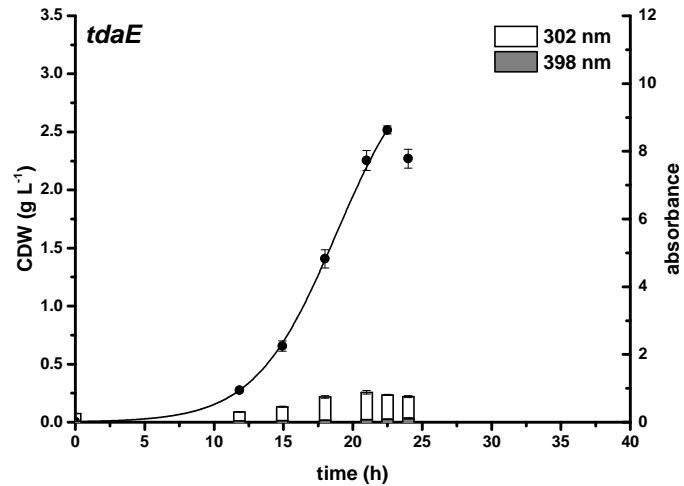

Supplement: S3 Fig — Shown are growth curves with corresponding absorption at 302 nm (TDA) and 398 nm (iron-complexed TDA) for the wild-type strain DSM 17395 and as controls for the mutant strains Δ262-kb and tdaE. (PDF) [file pone.0177295.s003.pdf]

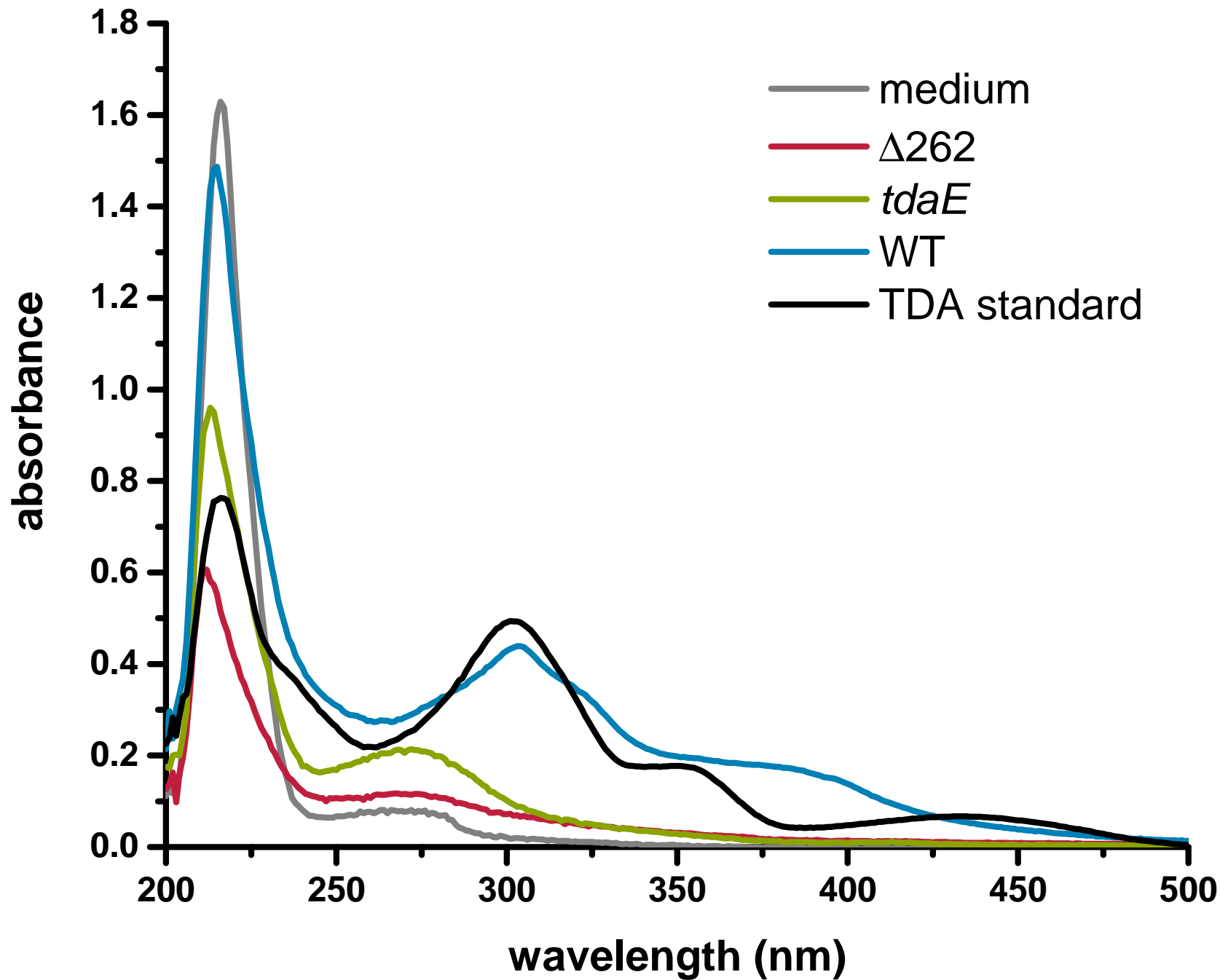

Supplement: S3 File — (PDF) [file pone.0177295.s006.pdf]
