# Supplementary material for: The limits to growth – energetic burden of the endogenous antibiotic tropodithietic acid in Phaeobacter inhibens DSM 17395
Source: PLoS One. 2017 May 8;12(5):e0177295. doi: 10.1371/journal.pone.0177295 (PMC5421792; doi:10.1371/journal.pone.0177295)
Supplement: S1 File — (PDF) [file pone.0177295.s004.pdf]

A: Authenticity of transposon mutants

PCR-assay of transposon mutants

Former plasmid curing experiments of *P. inhibens* DSM 17395 showed that the homogeneity of mutants might be impeded either by residual wild type cells that are attached to the mutant or by a heterogeneous plasmid population that still harbors genuine plasmids without the transposon insertion. Accordingly, we cultivated the transposon mutants in liquid Marine Broth (MB) medium with 120 µg/ml kanamycin (kan), plated the cultures under antibiotic selection on MB plates and streaked out single colonies on kan-plates three additional times for separation. Passaged mutants were grown in MB medium without antibiotics and total DNA was isolated with the DNeasy® Blood & Tissue Kit of QIAGEN. The absence of wild type cells and plasmids was documented with a sensitive PCR-assay.

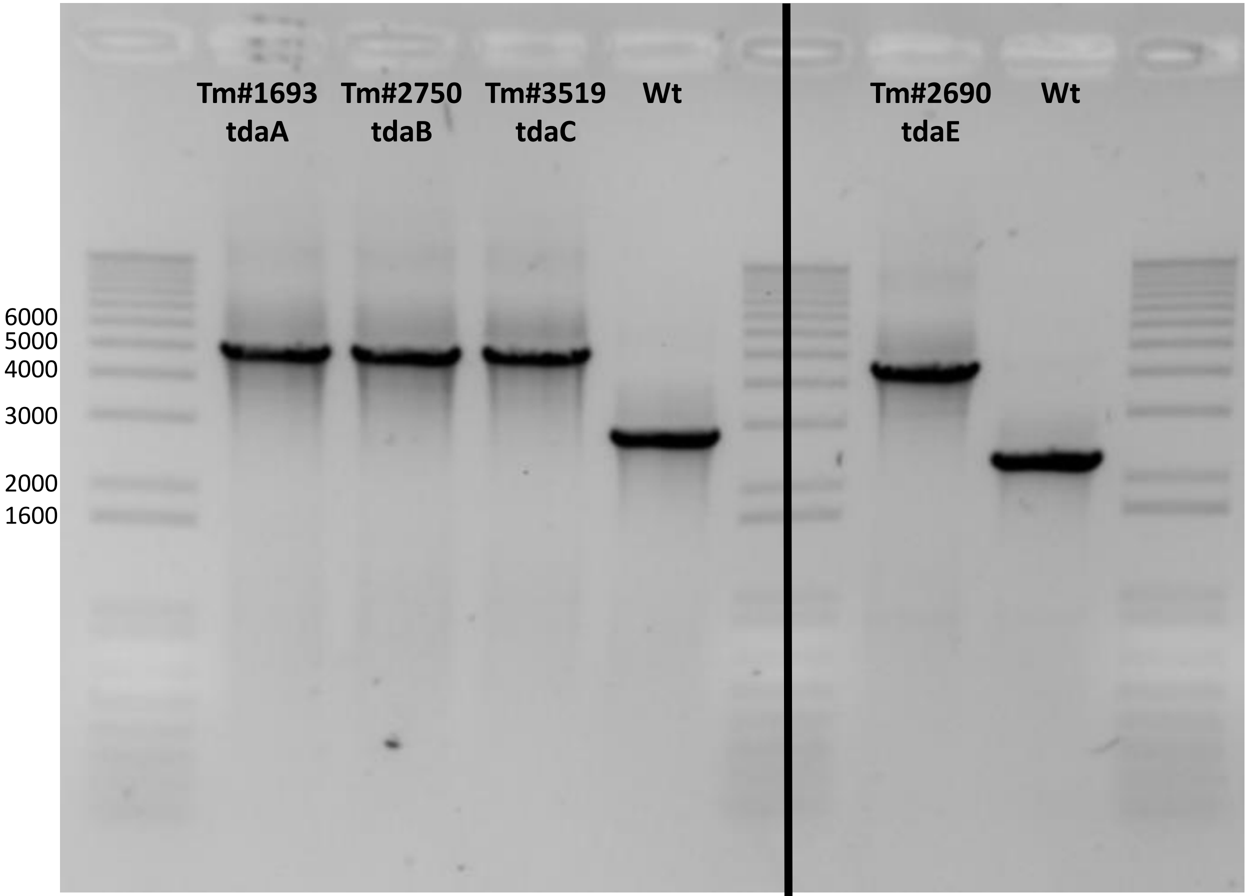

OneTaq Polymerase

initial denat.	94°C	30sec	
denat.	94°C	30 sec	x 30
annealing	56/57°C	30 sec	x 30
extension	68°C	4,5 min	x 30
final extension	68°C	10 min	

P1491	TCGCTATGACGCCACCTATGT
P1492	CCAACCAAAACCGCTCCATCC
Annealing temperature	56°C
Wildtype band	2787 bp
Mutant band	4788 bp

P1495	GTCAGGGCGCTTCGGTAAAAC
P1496	GGGAGTGCCATCAGCGTAAGC
Annealing temperature	57°C
Wildtype band	2249 bp
Mutant band	4250 bp

Conclusion

The PCR-assay documented the presence of the EZ-Tn5 transposon in the four TDA genes by a size shift of 2,001 bp compared to the respective band of the wild type. The sensitive test also unequivocally showed the absence of any residual wild type signal in the mutant strains and thus validated the integrity of the mutants.
