# Supplementary material for: The limits to growth – energetic burden of the endogenous antibiotic tropodithietic acid in Phaeobacter inhibens DSM 17395
Source: PLoS One. 2017 May 8;12(5):e0177295. doi: 10.1371/journal.pone.0177295 (PMC5421792; doi:10.1371/journal.pone.0177295)

TDA biosynthetic gene cluster of *P. inhibens* DSM 17395

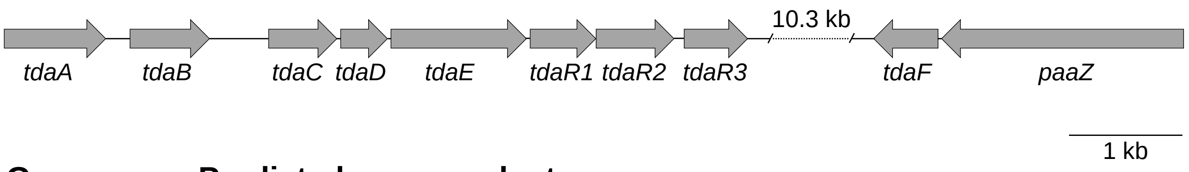

**Gene**	**Predicted gene products**
tdaA	Transcriptional regulator, LysR family
tdaB	Putative beta etherase
tdaC	Prephenate dehydratase TdaC-like protein
tdaD	Thioesterase superfamily protein
tdaE	Acyl-CoA dehydrogenase
*tdaR1**	Uncharacterized protein
*tdaR2**	Uncharacterized protein
*tdaR3**	Gamma-glutamylcyclotransferase
tdaF	Putative flavoprotein, HFCD family
paaZ	Phenylacetic acid degradation protein

*Wilson et al. (2016)

Proposed biosynthesis pathway of TDA by Brock et al. (2014)

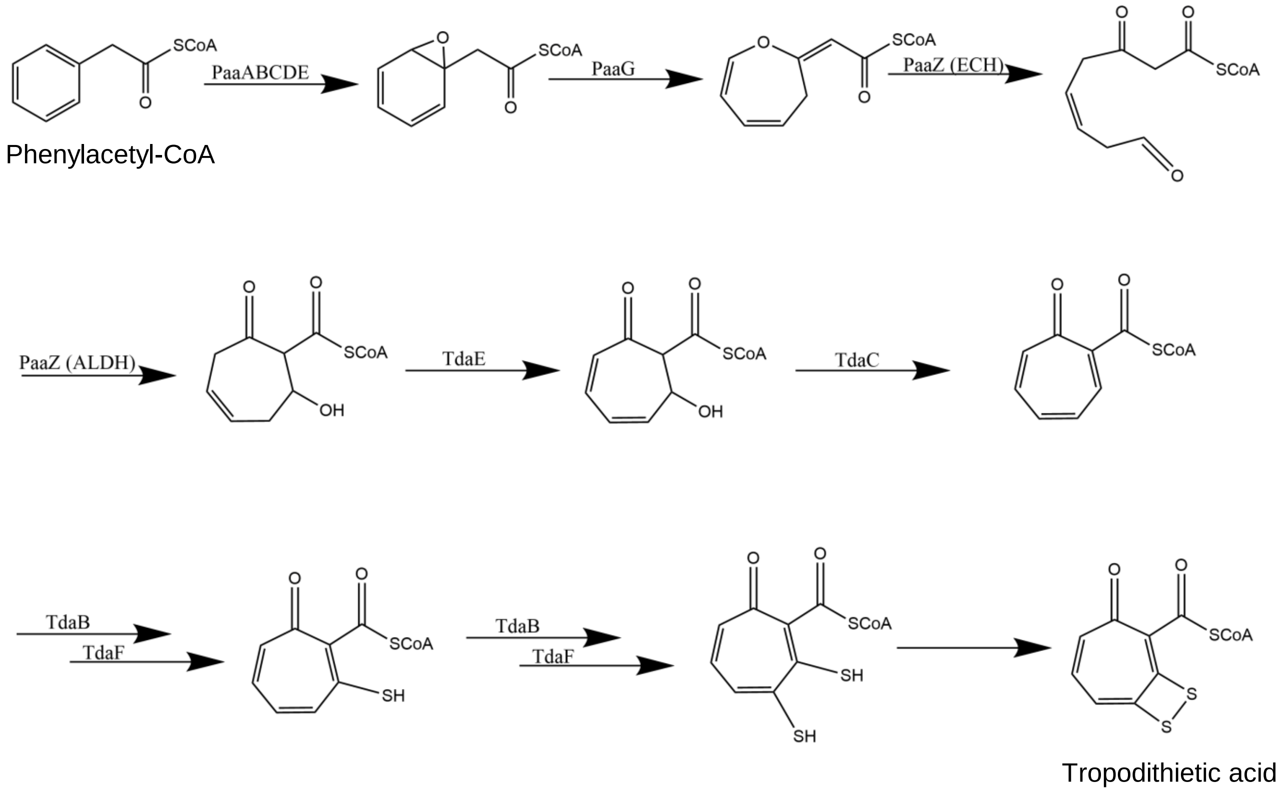

Supplement: S2 File — (PDF) [file pone.0177295.s005.pdf]
